# Recurrent Fever in Children

**DOI:** 10.3390/ijms17040448

**Published:** 2016-03-25

**Authors:** Sofia Torreggiani, Giovanni Filocamo, Susanna Esposito

**Affiliations:** 1 Pediatric Medium Intensive Care Unit, Department of Clinical Sciences and Community Health, Università degli Studi di Milano, Fondazione IRCCS Ca’ Granda Ospedale Maggiore Policlinico, 20122 Milan, Italy; sofia.torreggiani@unimi.it (S.T.); giovanni.filocamo@policlinico.mi.it (G.F.); 2 Pediatric Highly Intensive Care Unit, Department of Pathophysiology and Transplantation, Università degli Studi di Milano, Fondazione IRCCS Ca’ Granda Ospedale Maggiore Policlinico, 20122 Milan, Italy

**Keywords:** autoinflammatory disorders, pediatric infectious diseases, periodic fever, recurrent fever

## Abstract

Children presenting with recurrent fever may represent a diagnostic challenge. After excluding the most common etiologies, which include the consecutive occurrence of independent uncomplicated infections, a wide range of possible causes are considered. This article summarizes infectious and noninfectious causes of recurrent fever in pediatric patients. We highlight that, when investigating recurrent fever, it is important to consider age at onset, family history, duration of febrile episodes, length of interval between episodes, associated symptoms and response to treatment. Additionally, information regarding travel history and exposure to animals is helpful, especially with regard to infections. With the exclusion of repeated independent uncomplicated infections, many infective causes of recurrent fever are relatively rare in Western countries; therefore, clinicians should be attuned to suggestive case history data. It is important to rule out the possibility of an infectious process or a malignancy, in particular, if steroid therapy is being considered. After excluding an infectious or neoplastic etiology, immune-mediated and autoinflammatory diseases should be taken into consideration. Together with case history data, a careful physical exam during and between febrile episodes may give useful clues and guide laboratory investigations. However, despite a thorough evaluation, a recurrent fever may remain unexplained. A watchful follow-up is thus mandatory because new signs and symptoms may appear over time.

## 1. Introduction

Fever, defined as an elevation in core body temperature above normal values (≥37.9 °C) [1], is an extremely common presentation of a wide range of pathologies. Most frequently, acute fever is due to self-limited viral infections and uncomplicated bacterial infections and resolves within a week or less [2,3,4]. In a small number of cases, fever may be prolonged or reappear after a variable interval of apyrexia; these are source of deeper concern. Here, we focus on recurrent fever, which is characterized by febrile episodes that are separated by periods of normal temperature.

Although recurrent fever occurs frequently, representing 18%–42% of fevers of unknown origin in adults [5] and 69% in recent pediatric cases [6], it lacks a firm definition. In 1993, Knockaert *et al.* defined recurrent fever as a cyclical fever with seeming remission of the disease and fever-free intervals of at least two weeks [7]. To assess recurrence, other authors required at least three episodes of unexplained fever in a six-month period, with a minimum interval of seven days between episodes [8]; in other articles, the minimum interval was only 48 h [9,10]. In some cases, fever was considered recurrent if it consisted of multiple febrile episodes separated by an undefined interval of normal temperature. In this article, we use this last broad definition, only requiring the occurrence of at least three episodes of fever, thus excluding diseases in which fever has a biphasic course, such as poliomyelitis, leptospirosis, dengue fever, yellow fever, Colorado tick fever, and viral hemorrhagic fevers [11]. Although in a conspicuous proportion of cases, the cause of recurrent fever remains unknown [12], case history and physical findings can point toward the correct diagnosis. This article summarizes infectious and noninfectious causes of recurrent fever in pediatric age.

## 2. Differential Diagnosis of Recurrent Fever

The etiology of recurrent fever includes infectious and noninfectious causes, as summarized in Table 1. Among infectious causes, viral, bacterial, fungal, and parasitic diseases are included; among noninfectious causes there are immune-mediated and granulomatous diseases, periodic fever syndromes and autoinflammatory disorders, neoplasms, hypersensitivity diseases, and conditions with different etiologies that can be differentiated on the basis of clinical history and medical findings.

Fever periodicity and associated signs and symptoms may guide the pediatrician towards the correct diagnosis.

Recurrent fever occurring at irregular intervals may be caused by distinct illnesses involving different organ systems or by repeated unrelated infections of the same organ system (e.g., urinary tract). In some cases, recurrent fever may be the expression of a single illness in which fever and other signs and symptoms increase and decrease during the course of disease [13].

Sometimes, fever episodes present a regular “clockwork” periodicity, as in cyclic neutropenia and periodic fever, aphthous stomatitis, pharyngitis, and adenopathy syndrome (PFAPA syndrome); in other instances, the temporal pattern may be less regular and the child can appear in bad condition between the episodes without an adequate growth, as in auto-inflammatory disorders [14,15].

In some cases, hereditary autoinflammatory disorders (e.g., FMF and hyper-IgD syndrome) or EBV infection may cause fever at a regular interval and a good case history, clinical examination as well as specific laboratory tests may be helpful in confirming these diseases [8].

Neoplasms are not so common: leukemia and lymphoma are the two main cancers that can present with recurrent fever at admission and also for these diseases case history, clinical findings, and diagnostic tests appear useful for the confirmation of a specific diagnosis [9].

Table 2 and Table 3 summarize findings that are suggestive of some specific pathologies, showing differences between some autoinflammatory disorders, infectious diseases, and immune-mediated conditions.

## 3. Infectious Causes

When investigating recurrent fever, the most likely etiology to consider is infection. Especially in children under the age of six years, the most common cause of multiple febrile episodes is the occurrence of repeated upper respiratory tract infections (e.g., pharyngitis, otitis media). During infection, body temperature commonly fluctuates with brief apyretic intervals during the day, but the presentation of a single infectious disease with distinct febrile episodes separated by days of normal temperature is much less common. It can be useful to ask whether other family members or contacts exhibited the same symptoms. The frequency and features of the infections, failure to thrive and a family history of immune deficiency should be taken into account to exclude a possible immunodeficiency [16]. Table 4 illustrates the ten warning signs of primary immunodeficiency in children.

When the infections appear to be limited a single target organ (e.g., urinary tract, skin, lung), it is necessary to look for local predisposing conditions and refer the patient to the pertinent specialist.

More rarely, multiple febrile episodes are due to a single etiology. Fever and nonspecific symptoms and signs may be the presenting features of pathologies such as endocarditis, tuberculosis and chronic meningococcemia.

The main viral, bacterial, fungal and parasitic causes of recurrent fever are discussed in the next paragraphs. Some of these infections appear to be associated with a history of travel or animal contact.

### 3.1. Viral Diseases

Although repeated independent viral infections are the most common cause of recurrent fever in children as a consequence of their physiological susceptibility to infections, it is rare that a single viral disease is responsible for multiple febrile episodes.

Recurrent febrile episodes have been reported to be caused by EBV, Parvovirus B19 and HSV1 and HSV2 [8].

Fever associated with EBV infection lasts 7–10 days on average and usually resolves within three weeks; however, one case has been reported of a 15-year-old boy suffering from multiple episodes of fever, lymphadenopathy and occasional splenomegaly lasting 2–3 days at intervals of 2–3 weeks for a period of more than 10 years. In this patient, persistent active EBV infection was documented by serology, throat cultures and RNA *in situ* hybridization performed on the lymph nodes. No immune deficiency syndrome was identified [18]. The case described may be a mild expression of the rare Purtilo syndrome or severe chronic active EBV infection syndrome, which is characterized by persistent or intermittent fever, lymphadenopathy, hepatosplenomegaly and a peculiar EBV serology, with high titers of IgG anti-VCA and anti-EA in the absence of anti-EBNA [19].

In three cases, recurrent fever associated with arthralgia was attributed to persistent parvovirus B19 infection. Other accompanying symptoms included fatigue, night sweats, headache, abdominal pain, skin rash, hyperesthesia, swelling of the hands and feet, erythema nodosum, increased inflammatory markers and moderate anemia. Diagnosis was based on serologic and PCR tests [20,21].

HIV infection alone is not usually responsible for recurrent fever, but febrile illnesses often occur in patients with acquired immunodeficiency syndrome (AIDS) as a result of opportunistic infections.

### 3.2. Bacterial Diseases

Occult bacterial infection is a potential cause of recurrent fever. Infection may have a defined primary focus, as in urinary tract infections, cholangitis, endocarditis, osteomyelitis or dental abscesses [22,23]. Today, in countries where modern imaging techniques are available and appropriate antibiotic therapy is promptly prescribed, occult abscesses are rarely the cause of fevers of unknown origin in tertiary centers.

Endocarditis should always be suspected in the case of unexplained prolonged fever, even with a recurrent pattern, if a new cardiac murmur appears or the features of a preexisting one are modified. It should be noted that currently, new categories of children are at risk of infective endocarditis, such as critically ill patients with a normal heart structure and the presence of chronic indwelling catheters and children who have undergone surgical corrections for congenital cardiac disease. The emergence of these new at risk groups also has an impact on the etiology of endocarditis; viridans streptococci and *Staphylococcus aureus* remain the leading causes, but in the last few decades there has been a gradual increase in cases due to *Staphylococcus aureus*, with a decline in cases caused by streptococci [24].

Among specific agents identified as a cause of recurrent febrile episodes, relapsing fever due to borreliae, *Bartonella quintana*, *Mycobacterium tuberculosis*, *Spirillum minus*, meningococci in the setting of chronic meningococcemia and *Yersinia enterocolitica* must be mentioned.

#### 3.2.1. Relapsing Fever

Thus far, 23 validated *Borrelia* species that cause relapsing fever have been identified, but new ones have recently been described. All relapsing fever borreliae are vector-borne; *Borrelia recurrentis* is transmitted by a louse vector, while the other relapsing fever borreliae are transmitted by soft tick vectors, mostly *Ornithodoros* soft ticks. These soft ticks have a painless and unnoticeable bite and detach rapidly after a short blood meal, which is, however, sufficient for transmission. Humans are the reservoir of *Borrelia recurrentis* and *Borrelia duttonii*, while for the other relapsing fever borreliae, rodents are the main reservoir [25]. Poverty is a major risk factor for relapsing fever, but occupational contact with tick-infested environments and tourism in endemic regions are also associated with increased risk of infection [26,27,28,29]. *Borrelia recurrentis*, responsible for louse-borne relapsing fever (LBRF), was once cosmopolitan but is now mainly restricted to Africa in areas where the human body louse persists. Tick-borne relapsing fever (TBRF) is also common in Africa, as documented in recent reports from Senegal, Morocco and Togo [30,31,32] but is increasingly diagnosed in the United States as well, especially in western states. TBRF is among the top ten causes of mortality in children under the age of five in Tanzania [33]. Cases of relapsing fever have also been reported in the European Mediterranean region, South America and Asia [34].

Relapsing fever is characterized by recurrent fever, headache, myalgia, arthralgia, rigors and nausea. The cyclical pattern of fever is due to antigenic variation; each febrile episode corresponds to a change in the surface Vmp antigens. After 3–10 days of incubation, fever abruptly appears, normally resolving within 3–5 days. At the end of the primary febrile episode, rash over the trunk and shoulders may develop, disappearing in a couple of days. The first afebrile period lasts 2–7 days, while subsequent afebrile intervals usually lengthen as the disease progresses. In LBRF, the patient has 3–5 febrile episodes, while in untreated TBRF, up to 13 recurrences of fever are possible [25].

In LBRF, abdominal pain, hepatosplenomegaly, jaundice, renal involvement, central nervous system manifestations, thrombocytopenia and several bleeding manifestations have been described [35,36,37]. Death may occur due to hepatic or cardiac failure, pneumonia, subarachnoid hemorrhage or splenic rupture [25].

TBRF clinical presentation depends on the infecting *Borrelia* species, ranging from a mild to a severe febrile disease similar to LBRF.

Diagnosis relies on microscopy performed on a thick blood smear obtained during febrile episodes, animal inoculation, culture in specialized liquid media and molecular detection. A multiplex real-time PCR assay for the specific detection of *B. duttonii*/*recurrentis* and *B. crocidurae* has recently been developed [38].

#### 3.2.2. Trench Fever

Trench fever is due to *Bartonella quintana*, transmitted by the human body louse. It is commonly found in areas of high population density and poor hygienic conditions. Trench fever has a worldwide distribution; the only continent on which it is not found is Australia [39]. *Bartonella quintana* infection is re-emerging among the homeless populations in Europe and the United States.

Most commonly, the infection is characterized by a single febrile episode lasting 3–6 days, but recurrent fever may occur, with 3–8 episodes lasting 1–3 days each, with an afebrile interval of 4–6 days [40]. Associated findings include conjunctival injection, headache, tachycardia, myalgia, arthralgia and maculopapular rash on the trunk.

Diagnosis is based on serology, culture and molecular biology [41,42].

#### 3.2.3. Tuberculosis

Tuberculosis is a known cause of fever of unknown origin, being responsible for 5%–10% of cases in adult and pediatric patients [43,44,45,46,47,48]. A recurrent fever pattern has also been described, both in adults and children [47,49,50,51,52,53,54]. The duration of the febrile episodes ranges from a few hours to one week. Afebrile intervals may last 7–14 days.

Chest X-ray is of limited utility because tuberculosis in reported cases was often extrapulmonary, involving the pancreas, spleen, intestine, lymph nodes, or subcutaneous tissue. Tuberculin skin testing and interferon-gamma release assay are not always useful in confirming tuberculosis diagnosis, while computed tomography is of some help in detecting granulomas.

Cases of recurrent fever presumptively associated with tuberculosis infection were successfully treated with antituberculous therapy. When risk factors for tuberculosis are present, empirical antituberculous therapy should be considered in cases of unexplained recurrent fever [47].

#### 3.2.4. Chronic Meningococcemia

*Neisseria meningitidis* infection usually has a rapid onset and progression, but in some patients, chronic meningococcemia can develop. Chronic meningococcemia is characterized by recurrent fever, nontoxic appearance, arthralgia, headache, splenomegaly and a skin rash that usually consists of a purpuric and petechial papular eruption. Fever lasts approximately 12 h, with afebrile periods of 1–4 days [55]. This indolent presentation of meningococcal infection has been associated with host factors, such as abnormalities of the immune system [56,57,58,59], and pathogen factors, such as a reduced interleukin 6 inducing capacity and mutations in the *lpxL1* gene resulting in underacylated lipid A [60].

Diagnosis requires identification of *N. meningitidis* from blood cultures, which is easier in children than adults [61]. In the past, culturing skin lesions rarely allowed a microbiologic diagnosis, but new molecular pathogen-specific DNA amplification tests performed on suspicious skin lesions have become a useful tool. *N. meningitides*-specific DNA was detected in skin biopsy material from patients whose blood cultures were negative [62,63].

Complications are present in the minority of patients and include meningitis, endocarditis, nephritis, and septic arthritis [55]. It was described that steroid therapy may induce acute meningitis in patients affected by chronic meningococcemia [63,64].

Antibiotic treatment is effective. Overall, the prognosis for chronic meningococcemia is favorable; spontaneous remission is common in childhood [55]. With the implementation and diffusion of new anti-meningococcal vaccines, chronic meningococcemia may become an even rarer illness.

#### 3.2.5. Rat-Bite Fever

Rat-bite fever, caused by *Spirillum minus* (also known as Sodoku), may present with recurrent fever for a period of weeks or even months if not treated. Afebrile intervals usually last 3–7 days. When febrile episodes recur, they typically lose their intensity. The wound at the bite site at first heals spontaneously but reappears after 1–4 weeks of incubation when other clinical symptoms appear. Apart from aspecific symptoms such as fever, chills, headache and malaise, affected patients may present with enlargement of lymph nodes in the proximity of the bite wound, skin rash, arthritis, myalgia, diarrhea, vomiting, neuralgias and central nervous system symptoms. Complications include endocarditis, myocarditis, hepatitis and meningitis [65]. A history of contact with rats is fundamental to suspecting a rat bite fever diagnosis; a bite may be confirmed by direct dark-field microscopy of the wound exudate [66].

#### 3.2.6. Other Bacterial Causes of Recurrent Fever

Brucellosis has a wide range of clinical manifestations but usually presents with undulant fever, arthralgia or arthritis and hepatosplenomegaly. *Brucella* species, both *melitensis* and *suis*, were reported to be responsible for recurrent fever in adult patients [67,68,69]. It is important to investigate exposure to cattle, goats, other animals or raw milk approximately 1–4 weeks before clinical onset.

Recurrent fever associated with anemia, arthralgia and Genu Varum was identified as a late manifestation of congenital syphilis in a 28-month-old child [70].

Melioidosis due to *Burkholderia pseudomallei* is endemic in Southeast Asia and can present with multiple febrile episodes during a period of years [71]. Pediatric melioidosis usually presents as localized cutaneous disease in immunocompetent patients, but more severe cases have been described [72].

### 3.3. Fungal Diseases

Fungal infection is rarely a cause of recurrent fever, with histoplasmosis and coccidioidomycosis as possible etiologies.

Histoplasmosis was recognized as causing recurrent febrile episodes in adult immunocompromised patients [73] but should also be suspected in immunocompetent patients presenting with unexplained fever despite the fact that fever associated with histoplasmosis is most commonly prolonged and not recurrent in both adults and children [74,75]. In one pediatric immunocompetent patient, disseminated histoplasmosis was associated with intermittent fever lasting six months, bone pain, weight loss, lymphadenopathy and hepatosplenomegaly [76].

Coccidioidomycosis was reported to cause recurrent fever in adult patients and should be suspected also in children due to the increase in pediatric coccidioidomycosis cases [77,78].

### 3.4. Parasitic Diseases

Malaria, with its typical fever patterns, and visceral leishmaniasis are the parasitic causes of recurrent febrile episodes. In both pathologies, residence in endemic areas should guide clinical suspicion.

In one case, recurrent episodes of fever occurring at 1-, 3- and 6-weeks of age associated with neurological and ocular abnormalities were attributed to dysfunction of the hypothalamic thermoregulatory center, secondary to congenital toxoplasma infection [79].

#### 3.4.1. Malaria

Malaria is caused by the intracellular *Plasmodium* protozoa transmitted to humans by female *Anopheles* mosquitoes; the first species identified included *Plasmodium falciparum*, *Plasmodium malariae*, *Plasmodium vivax* and *Plasmodium ovale*. In 2004, a new species was recognized as capable of causing disease in humans, *Plasmodium knowlesi*, which is widespread mainly in Southeast Asian countries [80,81].

Malaria is characterized by paroxysms of fever, chills, sweats, fatigue and splenomegaly. Pallor is also common due to anemia. The typical fever bouts are due to the rupture of schizonts, which occurs every 48 h with *P. vivax* and *P. ovale* (tertian fever) and every 72 h with *P. malariae* (quartan fever). Periodicity is less strict at the beginning of the disease or in the case of *P. falciparum* and mixed infections. Regular tertian and quartan patterns are rarely observed in children [82]. *P. vivax* and *P. ovale* may persist in the liver as hypnozoites and cause clinical disease after months or years of well-being.

Due to its severity, malaria should be promptly ruled out in febrile patients who have recently traveled in endemic areas. Because paroxysms are less evident in children, who additionally present with more gastrointestinal symptoms compared with adults, the risk of misdiagnosis is high in the pediatric age group [83].

Diagnosis is established by identification of organisms on smears of peripheral blood. Identification of *P. knowlesi* and *P. malariae* requires specific PCR-based assays because they are morphologically indistinguishable, but in adults, *P. knowlesi* causes more severe disease [84]. It should be noted that *P. knowlesi* infection appears milder in children than adults [85].

#### 3.4.2. Visceral Leishmaniasis

Diagnosis of visceral leishmaniasis due to *Leishmania infantum* and *Leishmania donovani* should be considered in patients presenting with recurrent fever, abdominal discomfort, hepato-splenomegaly and pancytopenia if exposure to the sandfly is suspected [86,87]. Intermittent fever is typical during the first weeks or months of disease; only a minority of patients develops the complete clinical picture of marked hepato-splenomegaly and cachexia approximately six months after illness onset. Visceral leishmaniasis may be complicated by hemophagocytic lymphohistiocytosis [88,89].

The presence of amastigotes in bone marrow or tissue sections is diagnostic of visceral leishmaniasis [90]. PCR testing on peripheral blood is also possible but less sensitive and specific [91].

## 4. Noninfectious Causes

### 4.1. Immune-Mediated and Granulomatous Diseases

Among the common causes of recurrent fever, it is important to mention Crohn’s disease, especially in adolescents [92]. In Crohn’s disease, fever may precede the other typical manifestations of inflammatory bowel disease, such as abdominal discomfort or loose stools, by weeks or months [12]. Microcytic hypochromic anemia and growth retardation are useful diagnostic clues.

Behcet’s disease is a less common cause of recurrent fever, which should be included in the differential diagnosis with Crohn’s disease due to common clinical features [93]. Oral and genital ulcers, together with uveitis and skin lesions, are the main clinical manifestations of Behcet’s disease, ulcerative lesions may develop in any part of the gastrointestinal tract [94]. The age at onset in juvenile cases is usually between 8 and 12 years [95]. New classification criteria for pediatric Behçet’s disease have recently been proposed [96].

Additionally, systemic lupus erythematosus (SLE) and juvenile dermatomyositis (JDM) can be responsible for recurrent fever. Usually related signs and symptoms (e.g., characteristic skin involvement in JDM) and autoantibody testing (e.g., anti-dsDNA autoantibodies in SLE) strongly suggest the diagnosis.

The onset of autoimmune diseases in early childhood is rarer than in late childhood and adolescence but is, nonetheless, possible. In autoimmune diseases, fever episodes generally have a long duration, and during afebrile intervals, symptoms tend to persist, often worsening over time [97]. This chronic course helps differentiate autoimmune diseases from autoinflammatory disorders.

### 4.2. Periodic Fever Syndromes and Autoinflammatory Disorders

In periodic fever syndromes and autoinflammatory disorders, the recurrent febrile episodes are usually associated with the same predictable symptoms. In the interval between attacks, the child is generally in good health and grows well. Below details on the main history findings, signs, symptoms, and diagnostic criteria are reported.

#### 4.2.1. PFAPA Syndrome and Cyclic Neutropenia

If the periodicity is strictly regular, PFAPA syndrome and cyclic neutropenia should be suspected. Both pathologies are typically characterized by onset before the age of five years, a 21–28 day interval between febrile episodes, pharyngitis, stomatitis and cervical lymphadenopathy. The length of episodes is also similar: 3–6 days in PFAPA syndrome and 5–7 days in cyclic neutropenia. PFAPA syndrome is much more common than cyclic neutropenia, which should, nonetheless, be excluded by laboratory testing in all suspicious cases.

In PFAPA syndrome, blood tests show only mild leukocytosis and a moderate increase in erythrocyte sedimentation rate (ESR) during attacks, while no abnormality is found between episodes. A child affected by PFAPA syndrome has few complaints apart from recurrent fever and does not show an increased risk of infection [8,98].

On the contrary, a child with cyclic neutropenia may present repeated bacterial infections due to neutropenia. Cellulitis, especially in the perianal region, is common during the neutropenic period.

In cyclic neutropenia, neutropenia is not necessarily present at the time of fever; diagnosis requires multiple leukocyte counts, at least 2–3 per week for a 4–6 week period. Neutrophil counts lower than 500/µL are suggestive. Bone marrow examination at the time of neutropenia may confirm the diagnosis [99].

#### 4.2.2. Autoinflammatory Disorders

If the periodicity of fever episodes is not regular, an autoinflammatory disorder may be hypothesized. Autoinflammatory diseases are caused by a defect in the innate immune system, which leads to abnormally increased inflammation. Unlike in autoimmune diseases, in autoinflammatory disorders autoantibodies are not present and no association with human leukocyte antigen (HLA) class II genes has been found.

In monogenic or hereditary autoinflammatory disorders, in which there is often a family history of a similar disease, the cause of exaggerated inflammation is a mutation in one of the genes involved in innate immune pathways [100].

In monogenic autoinflammatory disorders, the onset of symptoms usually occurs in early childhood, although cases of FMF, TRAPS and FCAS have also manifested in adolescence or later [100]. Temperatures often exceed 39 °C, although in CAPS, low-grade fever is more typical [101]. In the interval between fever episodes, which is of variable duration, symptoms are only rarely present, but subclinical inflammation may easily be detected [97]. Triggering factors and associated signs and symptoms are helpful in the differential diagnosis. Additionally, ethnicity is relevant in some cases: FMF has a high incidence in patients with Mediterranean or Middle Eastern origin, while HIDS is more common in Dutch or Northern European patients. Features of the main monogenic autoinflammatory diseases presenting with recurrent fever are illustrated in Table 5.

Single cases of chronic atypical neutrophilic dermatosis with lipodystrophy and elevated temperature, deficiency of interleukin-36 receptor antagonist and NLPR12-associated fever syndrome have also been reported to present with recurrent fever [97].

A practical approach to the diagnosis of autoinflammatory diseases in childhood, which includes a probability score with the aim of optimizing genetic molecular analysis, has been proposed by Gattorno *et al.* [100,102].

Systemic JIA (sJIA) has recently been included in the group of non-monogenic autoinflammatory diseases due to its pathogenesis and clinical features [103,104].

In sJIA, fever can be the initial isolated sign for up to months. It is often associated with an evanescent salmon pink rash that transiently appears when the temperature increases and usually occurs in the late afternoon or evening, once or twice a day. The patient typically exhibits a rapid return to baseline or even below baseline body temperature [105]. In the first part of the disease course, the typical fever pattern may be less evident, but it may be observed after treatment with nonsteroidal anti-inflammatory drugs. Usually, the onset of symptoms occurs before 10 years of age, and fever is present for a period of weeks or even months [97]. Fever is the most common clinical presentation of sJIA, followed by arthritis and rash [106]. Arthritis may appear up to 10 years after the onset of systemic signs; therefore, diagnosis on the basis of International League of Associations for Rheumatology (ILAR) criteria for systemic JIA [107] can be delayed for a very long time. Other findings included in the classification criteria of sJIA are lymphadenopathy, hepatomegaly or splenomegaly and serositis.

Appearance of an unremitting fever in sJIA should be a red flag suggesting the possible development of a macrophage activation syndrome.

### 4.3. Other Causes of Recurrent Fever

In the differential diagnosis of recurrent fever, neoplasms should be ruled out, even if they are usually associated with prolonged fever [108]; only lymphoma, juvenile myelomonocytic leukemia and atrial myxoma have been reported to cause recurrent fever in children [109,110,111,112,113,114]. Leukemia and lymphoma are the two most common neoplasms that could present with recurrent fever.

An inflammatory pseudotumor was associated with recurrent fever in adults, while in children, only prolonged fever has been described [115,116].

Other rare causes of recurrent fever include factitious fever, drug fever, diabetes insipidus, histiocytic disorders and central nervous system abnormalities, such as agenesis of the corpus callosum or hypothalamic dysfunction [8].

## 5. Conclusions

Most children with recurrent fever have self-limited, common illnesses due to the physiological susceptibility to infections typical of the pediatric age group and will have a favorable prognosis. In a minority of cases, the cause of recurrent fever is a more rare disease that requires second line investigations and specific treatment.

When investigating recurrent fever, it is important to consider the age at onset, family history, duration of febrile episodes, length of the interval between episodes, associated symptoms and response to treatment. Additionally, knowledge of travel history and exposure to animals is helpful, especially with regard to infections. With the exception of repeated independent uncomplicated infections, many infective causes of recurrent fever are relatively rare in Western countries and a travel history in endemic areas is extremely helpful in supporting the diagnosis; therefore, clinicians should be attuned to suggestive case history data. It is important to rule out the possibility of an infectious process or a malignancy, especially if steroid therapy is being considered. After excluding an infectious or neoplastic etiology, immune-mediated and autoinflammatory diseases should be taken into consideration.

Together with case history data, a careful physical exam during and between febrile episodes may provide useful clues and guide laboratory investigations. However, despite thorough evaluation, recurrent fever may remain unexplained. A watchful follow-up is thus mandatory because new signs and symptoms may appear over time.

## Figures and Tables

**Table 1:** Etiology of recurrent fever in children.

Infectious Causes	Noninfectious Causes
**Viral diseases:** Repeated independent respiratory viral infections; Parvovirus B19 infection; Epstein-Barr (EBV) virus infection; Recurrent herpes virus infection. **Bacterial diseases:** Relapsing fever (*Borrelia recurrentis* and other borreliae); Brucellosis; Trench fever (*Bartonella quintana*); Syphilis (*Treponema pallidum*); Rat bite fever (*Spirillum minus*); Melioidosis (*Burkholderia pseudomallei*); Whipple disease; Chronic meningococcemia; Infective endocarditis; Subacute cholangitis; Abscesses, especially occult dental abscesses; Osteomyelitis; Tuberculosis. **Fungal diseases:** Histoplasmosis; Coccidioidomycosis. **Parasitic diseases:** Malaria; Visceral leishmaniasis.	**Immune-mediated and granulomatous diseases:** Crohn disease; Behçet disease; Systemic lupus erythematosus (SLE); Juvenile dermatomyositis (JDM); Acute rheumatic fever; Leukoclastic angiitis syndromes; Sarcoidosis; Granulomatous hepatitis. **Periodic fever syndromes and autoinflammatory disorders:** Cyclic neutropenia; Periodic fever; aphthous stomatitis; pharyngitis; adenopathy (PFAPA) syndrome; Familial Mediterranean fever (FMF); Hyperimmunoglobulinemia D with periodic fever syndrome (HIDS); Cryopyrin-associated periodic syndromes (CAPS): familial cold autoinflammatory syndrome (FCAS), Muckle-Wells syndrome (MWS), Neonatal onset multisystem inflammatory disease (NOMID); TNF receptor-associated periodic syndrome (TRAPS); Systemic juvenile idiopathic arthritis (sJIA); **Neoplasms;** **Hypersensitivity diseases:** Hypersensitivity pneumonitis; Drug fever; Weber-Christian disease (panniculitis). **Other conditions:** Sweet syndrome; Fabry disease; Congenital insensitivity to pain with anhidrosis; Anhidrotic ectodermal dysplasia; Sickle cell crisis; Castleman disease; Erdheim-Chester disease; Kikuchi-Fujimoto disease; Diabetes insipidus; Central nervous system abnormalities; Factitious fever.

**Table 2:** Case history findings suggestive of a specific diagnosis.

History Findings	Possible Diagnoses
Attack provoked by cold exposure	FCAS
Attack after immunizations	HIDS
Rat exposure	Rat bite fever
Cattle or raw milk exposure	Brucellosis
Travel in endemic areas *	Malaria, Relapsing fever, Visceral leishmaniasis

FCAS: familial cold autoinflammatory syndrome; HIDS: hyperimmunoglobulinemia D with periodic fever syndrome. * Malaria endemic areas: Africa, Middle East, Central and South America, Southeast and Western Pacific region; relapsing fever endemic areas: Africa, Western United States, Mexico, Central and South America, Mediterranean region, Central Asia; visceral leishmaniasis endemic areas: Indian subcontinent, East Africa, Brazil, Southern Europe.

**Table 3:** Signs and symptoms suggestive of a specific diagnosis.

Signs and Symptoms	Possible Diagnoses
Relative bradycardia	Brucellosis, drug fever
Bradycardia due to a conduction defect	Acute rheumatic fever, Infective endocarditis
Oral ulcers	Crohn disease, Behçet disease, Cyclic neutropenia, PFAPA, HIDS, HSV, Drug fever
Lymphadenopathy	Cyclic neutropenia, PFAPA, sJIA, HIDS, EBV
Arthritis/arthralgia	sJIA, FMF, HIDS, TRAPS, MWS, FCAS, Behçet disease, Parvovirus B19, Relapsing fever, Trench fever, Chronic meningococcemia, Rat bite fever, Brucellosis
Splenomegaly	sJIA, HIDS, FMF, TRAPS, EBV, Relapsing fever, Chronic meningococcemia, Brucellosis, Malaria, Visceral leishmaniasis
Uveitis	Crohn disease, Behçet disease
Weight loss	Crohn disease, malignancy
Fatigue	Endocarditis, sJIA, malignancy
Abdominal pain	Crohn disease, FMF, HIDS, TRAPS, Parvovirus B19, Relapsing fever
Serositis	FMF, sJIA, Systemic lupus erythematosus
Conjunctivitis	TRAPS (painful conjunctivitis), FCAS, Trench fever
Genital ulcers	Behçet disease
Transient rash during fevers	sJIA
Petechiae	Chronic meningococcemia
Erythema nodosum	Crohn disease, Behçet disease, Parvovirus B19
Erysipelas-like erythema	FMF
Sensorineural deafness	MWS, NOMID
Morning fever	Diabetes insipidus

PFAPA: Periodic fever, aphthous stomatitis, pharyngitis, adenopathy syndrome; FMF: Familial Mediterranean fever; HIDS: Hyperimmunoglobulinemia D with periodic fever syndrome; FCAS: familial cold autoinflammatory syndrome; MWS: Muckle-Wells syndrome; NOMID: Neonatal onset multisystem inflammatory disease; TRAPS: TNF receptor–associated periodic syndrome; sJIA: systemic juvenile idiopathic arthritis; HSV: Herpes simplex virus. EBV: Epstein-Barr virus.

**Table 4:** Warning signs of primary immunodeficiency in children.

Warning Signs of Primary Immunodeficiency in Children
Four or more episodes of otitis within 1 year
Two or more serious episodes of sinusitis within 1 year
Two or more cases of pneumonia within 1 year
Failure to gain weight or grow normally
Recurrent, deep skin or organ abscesses
Two or more deep-seated infections including septicemia
Persistent thrush in mouth or fungal infection on skin
Two or more months on antibiotics with little effect
Need for intravenous antibiotics to clear infections
A family history of primary immunodeficiency

Adapted from the Jeffrey Modell Foundation [17].

**Table 5:** Monogenic autoinflammatory diseases presenting with recurrent fever.

Disease	Gene Defect and Inheritance	Age at Onset	Duration of Febrile Episodes	Main Associated Findings
FMF	MEFV	First years of life	12–72 h	Abdominal pain, thoracic pain, arthritis.
	AR			
HIDS	MVK	First year of life	3–7 days	Abdominal pain, diarrhea, hepatosplenomegaly, lymphadenopathy.
	AR			
FCAS	NLRP3	First year of life	12 h–2 days	Urticarial rash induced by cold, arthralgia, conjunctivitis.
	AD			
MWS	NLRP3	Childhood	2–3 days, if present	Urticarial rash, sensorineural deafness.
	AD			
NOMID	NLRP3	Neonatal	Variable, if present	Rash, neurologic symptoms, skeletal abnormalities.
	AD (sporadic)			
TRAPS	TNFRSF1A	First year of life	1–3 weeks	Arthromyalgia, fasciitis, rash, conjunctivitis and periorbital edema, splenomegaly.
	AD			

AR: Autosomal recessive; AD: Autosomal dominant; FMF: Familial Mediterranean Fever; HIDS: Hyperimmunoglobulinemia D with periodic fever syndrome; FCAS: familial cold autoinflammatory syndrome; MWS: Muckle-Wells syndrome; NOMID: Neonatal onset multisystem inflammatory disease; TRAPS: TNF receptor–associated periodic syndrome; sJIA: systemic juvenile idiopathic arthritis.

## Abbreviations

EBV	Epstein-Barr virus
FCAS	familial cold autoinflammatory syndrome
FMF	familial Mediterranean fever
HIDS	hyperimmunoglobulinemia D with periodic fever syndrome
HSV	herpes simplex virus
JDM	juvenile dermatomyositis
MWS	Muckle-Wells syndrome
NOMID	neonatal onset multisystem inflammatory disease.
PFAPA	periodic fever, aphthous stomatitis, pharyngitis, adenopathy syndrome
sJIA	systemic juvenile idiopathic arthritis
SLE	systemic lupus erythematosus
TRAPS	TNF receptor–associated periodic syndrome
